# The effect of glucose dynamics on plasma copeptin levels upon glucagon, arginine, and macimorelin stimulation in healthy adults: Data from: Glucacop, Macicop, and CARGO study

**DOI:** 10.1007/s11102-022-01240-0

**Published:** 2022-06-20

**Authors:** Cihan Atila, Sophie Monnerat, Sandrine Andrea Urwyler, Julie Refardt, Bettina Winzeler, Mirjam Christ-Crain

**Affiliations:** 1 Departments of Endocrinology, Diabetology and Metabolism, University Hospital Basel, Petersgraben 4, 4031 Basel, Switzerland; 2 Department of Clinical Research, University of Basel, Basel, Switzerland

**Keywords:** Copeptin, Glucagon, Macimorelin, Arginine, Pituitary stimulation test

## Abstract

**Purpose:**

Non-osmotic stimulation tests using glucagon, arginine, or macimorelin were recently evaluated for their ability to assess posterior pituitary function. Glucagon and arginine, but not macimorelin, stimulated copeptin secretion (a surrogate marker of vasopressin) and, therefore, provide novel tests to assess the posterior pituitary. The exact underlying mechanism behind their stimulatory effect remains elusive.

**Methods:**

This analysis combined data from three diagnostic studies conducted at the University Hospital Basel, Switzerland. In total, 80 healthy adults underwent the glucagon (n = 22), arginine (n = 30), or macimorelin (n = 28) stimulation tests. The primary objective was to investigate glucose course upon glucagon, arginine, and macimorelin stimulation tests and its effect on plasma copeptin release.

**Results:**

Upon glucagon stimulation, the median [IQR] glucose level at baseline was 5.0 [4.6, 5.2] mmol/l, peaked at 8.1 [7.2, 9.4] mmol/l after 30 min and decreased to a minimum of 3.8 [3.5, 4.5] mmol/l after 120 min. The median copeptin increase upon glucagon stimulation was 7.7 [2.6, 28.0] pmol/l. Upon arginine, the glucose level at baseline was 4.9 [4.8, 5.5] mmol/l, peaked at 6.0 [5.2, 6.4] mmol/l after 30 min and decreased to a minimum of 4.3 [3.8, 4.8] mmol/l after 60 min. The median copeptin increase upon arginine stimulation was 4.5 [2.9, 7.5] pmol/l. Upon macimorelin, glucose levels showed no notable dynamics over the 120 min, and no major change in copeptin was observed.

In the pooled dataset, a decrease in glucose levels was significantly correlated with copeptin increase (ρ = 0.53, p < 0.01).

**Conclusion:**

A similar course in plasma glucose was observed in the copeptin-stimulating test, i.e., after glucagon and arginine, while macimorelin had no effect on glucose and copeptin levels. We hypothesize that a drop in glucose levels observed upon glucagon and arginine might stimulate copeptin.

## Introduction

Arginine vasopressin (AVP) is a peptide hormone produced in the paraventricular and supraoptic nuclei of the hypothalamus and released into the circulation from the posterior pituitary gland [1]. AVP acts mainly in the collecting duct of the kidneys via upregulation of aquaporin-2 channels resulting in free water reabsorption [1]. Physiologically, AVP and copeptin, the C-terminal segment of the AVP precursor peptide, [2, 3] are secreted either after an osmotic stimulus, i.e., increased plasma osmolality, or upon non-osmotic stimuli, such as decrease in effective arterial blood volume or unspecific stress, nausea, pain or hypoglycemia [4]. For example, an insulin-hypoglycemia test with a glucose level below 2 mmol/L resulted in an almost threefold increase in copeptin levels in healthy adults [5].

Recently, non-osmotic tests such as glucagon, arginine, or macimorelin (a ghrelin receptor agonist) stimulations were evaluated in the differential diagnosis of diabetes insipidus [6–8]. Results showed that glucagon and arginine, but not macimorelin, stimulate copeptin secretion and, therefore, provide non-osmotic stimulation tests to assess the posterior pituitary function [9, 10]. The exact underlying mechanism behind the stimulatory effect of glucagon and arginine on the posterior pituitary and the differences to macimorelin remains elusive. Since glucose dynamics, as observed after insulin-induced hypoglycemia, seem to trigger copeptin secretion, we hypothesized that the difference in glucose dynamics might explain the differences in copeptin release between stimulating tests (arginine and glucagon) and the non-stimulating test (macimorelin).

In this current analysis, we therefore investigated glucose dynamics upon glucagon, arginine, and macimorelin stimulation tests and its effect on plasma copeptin release in healthy adults.

## Materials and methods

### Study design and participant

This study combined data from three prospective diagnostic studies conducted at the University Hospital Basel, Switzerland. In brief, 22, 30 and 28 healthy participants underwent the glucagon, arginine, and macimorelin stimulation test, respectively. Healthy adults were eligible if they were aged 18 years or older with a body mass index between 18.5 and 25 kg/m^2^, had normal drinking habits, and no history of polyuria. Exclusion criteria were a hemoglobin level < 120 g/l, pregnancy, or breastfeeding. Full details of the studies’ rationale, design, test protocol, and statistical analysis have been published elsewhere [9–11]. The local ethics committee approved the study protocols. Written informed consent was obtained from all study participants. The studies were registered on ClinicalTrials.gov, identifier NCT04550520, NCT01879137, NCT03844217.

### Glucagon test protocol

Healthy participants presented between 08:00 and 10:00 a.m. after an overnight fast of 8 h and fluid restriction of 2 h for two test days in random order, one test day undergoing subcutaneous injection of 1 mg glucagon and one test day undergoing 1 ml subcutaneous injection of a placebo, i.e., 0.9% sodium chloride. All participants received 8 mg Ondansetron 10 min before the test to prevent nausea. A catheter was placed into an antecubital vein. A first blood sample was collected at baseline, and glucagon 1 mg (Glucagen® NovoNordisk—Hypokit) or placebo (0.9% sodium chloride) was injected. Blood was then collected at 30, 60, 90, 120, 150 and 180 min for analysis of plasma copeptin and glucose. Participants were not allowed to drink during the test.

### Arginine infusion test protocol

Healthy participants presented between 08:00 and 10:00 a.m. after an overnight fast of 8 hours and fluid restriction of 2 hours. A catheter was placed into an antecubital vein. A first blood sample was collected at baseline, before the infusion of arginine (l­arginine­hydrochloride 21%, Braun, B Braun Melsungen AG, Melsungen, Germany), at a dose of 0.5 g/kg bodyweight diluted in 500 ml of 0.9% sodium chloride solution, was started and infused over 30 min. Blood was collected at 30, 45, 60, 90, and 120 min for the analysis of plasma copeptin and glucose. Participants were not allowed to drink or eat during the test.

### Macimorelin test protocol

Healthy participants presented between 08:00 and 10:00 a.m. after an overnight fast of 8 hours and fluid restriction of two test days at the study site. A catheter was placed into an antecubital vein. A first blood sample was collected at baseline, before participants received macimorelin orally, the first test day with a dose of 0.50 mg/kg body weight and the second test day with a dose of 0.75 mg/kg body weight with a wash-out period of at least 1 week in between. Blood then was collected at 30, 45, 60, 90, and 120 min for analysis of plasma copeptin and glucose. Participants were not allowed to drink during the test.

### Laboratory measurements

Serum samples for plasma copeptin and EDTA-blood samples for glucose were immediately centrifuged at 4 °C and stored at − 80 °C until central batch analysis. Plasma copeptin concentration upon glucagon and arginine stimulation was measured in one batch, and upon macimorelin stimulation immediately after sampling. Plasma copeptin was measured with a commercial automated immunofluorescence assay (B.R.A.H.M.S Copeptin-proAVP KRYPTOR, Thermo Scientific Biomarkers, Hennigsdorf, Germany). Growth hormone levels were only measured upon the glucagon and macimorelin test. Plasma growth hormone was measured with an electrochemiluminescence immunoassay (ECLIA) (Cobas8000, Roche Diagnostics GmbH, Mannheim, Germany).

### Statistical analysis

The primary outcome was the correlation between the maximal decrease in plasma glucose levels and maximal increase in copeptin level upon glucagon, macimorelin, and arginine stimulation tests.

To derive the primary outcome i.e., a decrease in glucose and an increase in copeptin for each participant and provocation test, we determined the maximal glucose, copeptin, and growth hormone value measured between baseline and the end of each stimulation (glucose_max,_ copeptin_max,_ growth hormone_max,_) and subtracted the corresponding minimum glucose, copeptin, and growth hormone value measured upon each test (glucose_min,_ copeptin_min,_ growth hormone_min_). The correlations are presented visually with scatterplots, and glucose/copeptin dynamics after each provocation test are presented visually with boxplots. All analyses were performed in R version 4.0.3 (2020-10-10). Hypothesis testing was two-sided, and p values < 0.05 were considered statistically significant.

## Results

### Baseline characteristics

In total 80 healthy participants were included. The median age was similar in the glucagon and macimorelin cohort, whereas participants in the arginine cohort were slightly older. The body mass index (BMI) was balanced between the three groups, participants in the glucagon cohort were predominantly male. Baseline characteristics for each group are presented in Table 1.

**Table 1:** Baseline characteristics

	Overall	Glucagon	Arginine	Macimorelin
Number of participants	80	22	30	28
Age in years	26 [25, 30]	25 [22, 29]	30 [27, 37]	25 [25, 27]
Female sex n (%)	42 (52)	10 (45)	17 (57)	15 (54)
BMI (kg/m^2^)	22.3 [20.9, 24.6]	22.2 [21.5, 23.9]	23.3 [20.5, 26.0]	22.3 [21.5, 24.4]
Contraceptive medication n (%)	12 (15)	1 (5)	6 (20)	5 (18)
Current smoker n (%)	15 (19)	5 (23)	10 (33)	0 (0)

Continuous variables are presented as median [IQR], and categorical variables are presented as frequency (percentage)

### Glucose and copeptin dynamics upon glucagon, macimorelin, and arginine stimulation

**Fig. 1:**
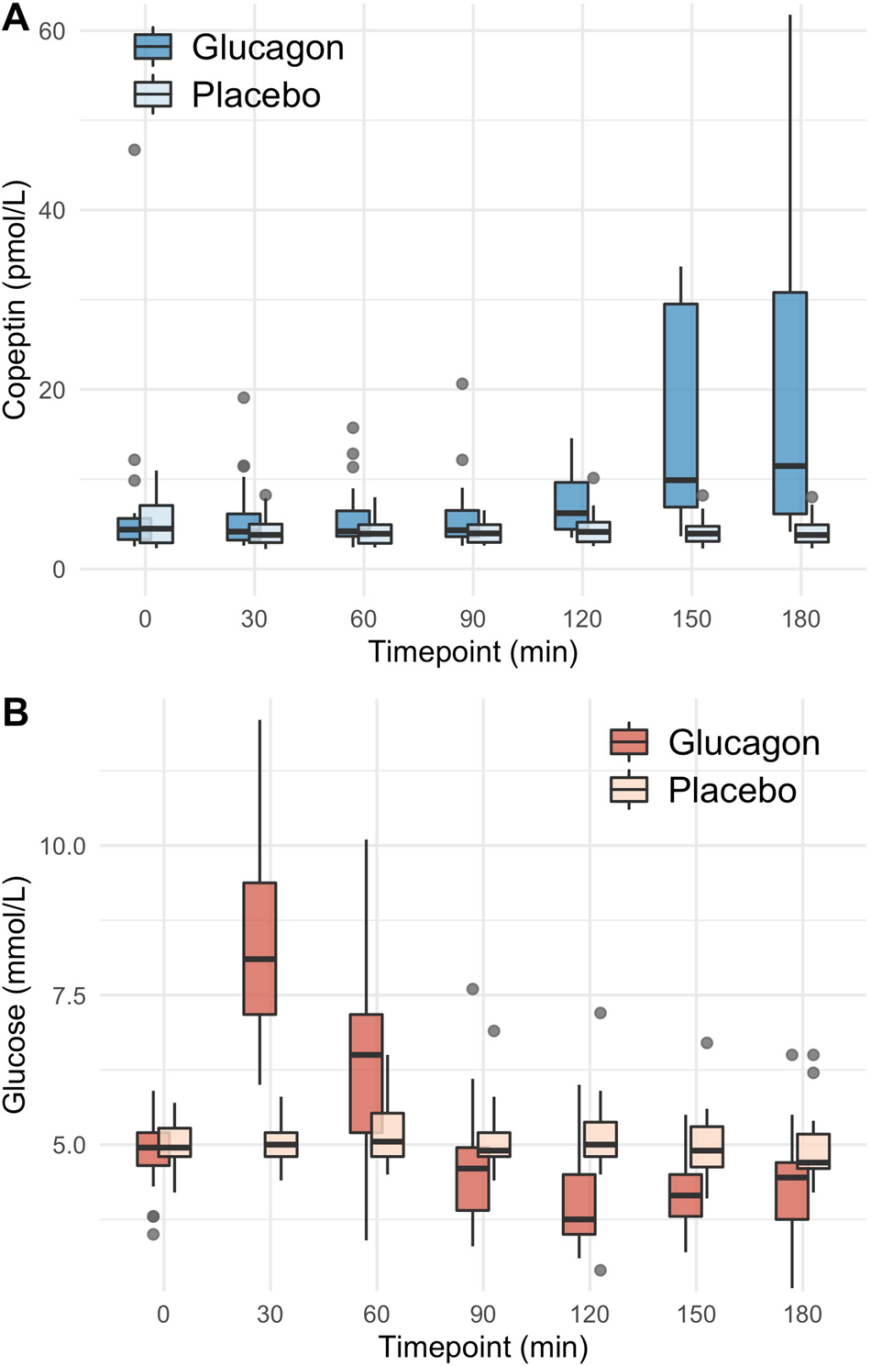
**a**/**b** Change in plasma copeptin and glucose levels upon glucagon stimulation test. Plasma copeptin levels in pmol/l and glucose levels in mmol/l within 180 min after glucagon and placebo stimulation. Boxes span the interquartile range [IQR]; the thick horizontal line is the median. Whiskers are the most extreme values lying within the box edge and 1.5 times the IQR. All other values are considered to be outliers and plotted as individual points

Upon glucagon stimulation, the median [IQR] glucose level at baseline was 5.0 [4.6, 5.2] mmol/l, peaked at 8.1 [7.2, 9.4] mmol/l after 30 min and decreased to a minimum of 3.8 [3.5, 4.5] mmol/l after 120 min, resulting in a difference between maximum and minimum glucose level of 4.4 [3.6, 6.1] mmol/l (Fig. 1a, Table 2). The median copeptin at baseline was 4.4 [3.3, 5.6] pmol/l and increased to a maximum of 11.5 [6.1, 30.8] pmol/l at 180 min after glucagon stimulation, resulting in a difference between baseline and maximum copeptin of 7.7 [2.6, 28.0] pmol/l (Fig. 1b, Table 2). After glucagon injection, the median time from baseline to minimum glucose level was at 120 min and to the maximum copeptin level was at 150 min.

**Table 2:** Glucose, copeptin, and growth hormone levels upon glucagon, arginine, and macimorelin

	0 min	30 min	45 min	60 min	90 min	120 min	150 min	180 min	delta
Glucagon^A^									
Glucose in mmol/l	5.0 [4.6, 5.2]	8.1 [7.2, 9.4]	*NA*	6.5 [5.2, 7.2]	4.6 [3.9, 5.0]	3.8 [3.5, 4.5]	4.2 [3.8, 4.5]	4.4 [3.8, 4.7]	4.4 [3.6, 6.1]
Copeptin in pmol/l	4.4 [3.3, 5.6]	4.2 [3.2, 6.1]	*NA*	4.2 [3.6, 6.5]	4.3 [3.6, 6.5]	6.2 [4.4, 9.6]	9.9 [6.9, 29.5]	11.5 [6.1, 30.8]	7.7 [2.6, 28.0]
Growth hormone in mlU/l	7 [1, 14]	4 [2, 11]	*NA*	2 [1, 6]	2 [1, 4]	25 [4, 42]	45 [22, 77]	32 [12, 53]	46 [29, 88]
Arginine									
Glucose in mmol/l	4.9 [4.8, 5.5]	6.0 [5.2, 6.4]	5.4 [4.5, 5.8]	4.3 [3.8, 4.8]	4.4 [3.9, 4.9]	4.8 [4.5, 5.1]	*NA*	*NA*	1.9 [1.2, 2.3]
Copeptin in pmol/l	5.2 [3.3, 10.9]	8.8 [5.1, 15.5]	9.0 [5.3, 16.8]	9.8 [6.4, 19.6]	9.8 [6.8, 19.4]	8.7 [6.1, 20.1]	*NA*	*NA*	4.5 [2.9, 7.5]
Macimorelin^B^									
Glucose in mmol/l	5.0 [4.8, 5.4]	5.2 [4.9, 5.5]	5.2 [4.9, 5.6]	5.2 [5.0, 5.5]	5.2 [4.9, 5.6]	5.1 [4.8, 5.4]	*NA*	*NA*	0.6 [0.4, 1.1]
Copeptin in pmol/l	4.2 [3.5, 6.2]	3.5 [3.0, 5.1]	3.8 [3.1, 5.5]	3.6 [2.9, 5.6]	3.9 [2.9, 5.5]	3.7 [2.9, 5.0]	*NA*	*NA*	0.1 [-0.4, 0.6]
Growth hormone in mlU/l	9 [0, 20]	47 [20, 89]	111 [75, 128]	91 [64, 116]	62 [39, 82]	32 [20, 52]	*NA*	*NA*	76 [61, 95]

Continuous variables are presented as median [IQR]

*NA* not available

^A^Data for the placebo stimulation are not presented

^B^Only data for the high dose macimorelin stimulation are presented

**Fig. 2:**
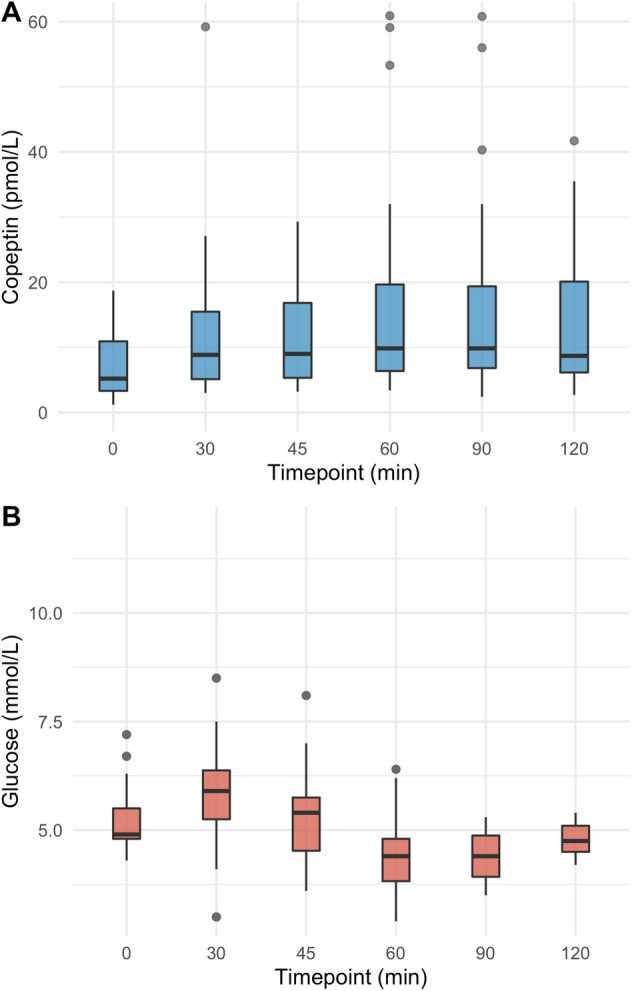
**a**/**b** Change in plasma copeptin and glucose levels upon arginine stimulation test. Plasma copeptin levels in pmol/l and glucose levels in mmol/l within 120 min arginine stimulation. Boxes span the interquartile range [IQR]; the thick horizontal line is the median. Whiskers are the most extreme values lying within the box edge and 1.5 times the IQR. All other values are considered to be outliers and plotted as individual points

Upon arginine stimulation, the glucose level at baseline was 4.9 [4.8, 5.5] mmol/l, peaked at 6.0 [5.2, 6.4] mmol/l after 30 min and decreased to a minimum of 4.3 [3.8, 4.8] mmol/l after 60 min, resulting in a difference between maximum and minimum glucose level of 1.9 [1.2, 2.3] mmol/l (Fig. 2a, Table 2). The median copeptin at baseline was 5.2 [3.3, 10.9] pmol/l and increased to a maximum of 9.8 [6.4, 19.6] pmol/l at 60 min, resulting in a difference between baseline and maximum copeptin of 4.5 [2.9, 7.5] pmol/l (Fig. 2b, Table 2). After arginine infusion, the median time from baseline to the minimum glucose level and to the maximum copeptin level was simultaneously at 60 min.

**Fig. 3:**
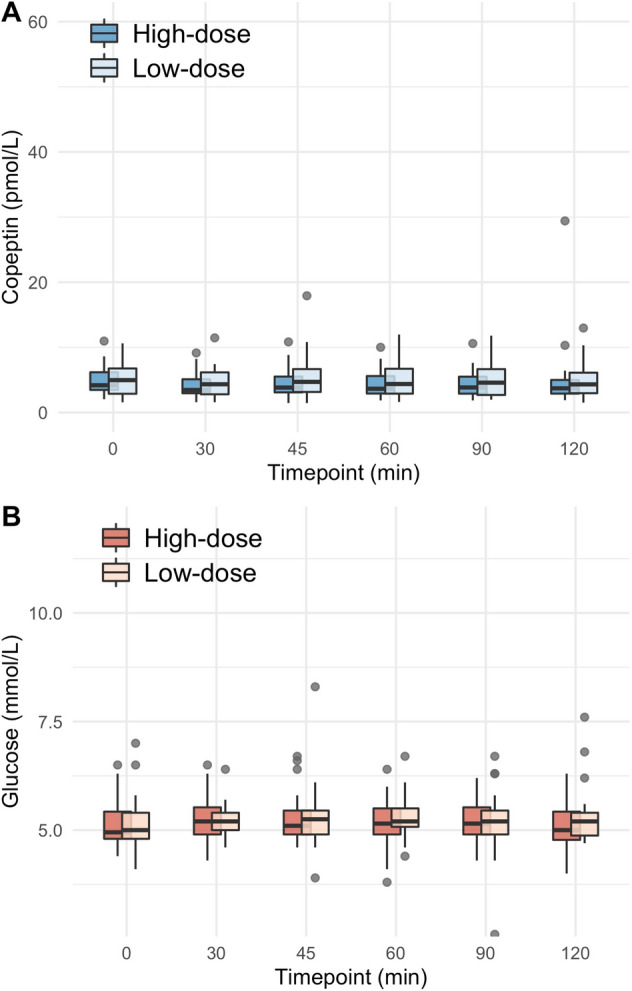
**a**/**b** Change in plasma copeptin and glucose levels upon macimorelin stimulation test. Plasma copeptin levels in pmol/l and glucose levels in mmol/l within 120 min after macimorelin stimulation (low dose, i.e., 0.5 g/kg bodyweight; high dose, i.e., 0.75 g/kg body weight). Boxes span the interquartile range [IQR]; the thick horizontal line is the median. Whiskers are the most extreme values lying within the box edge and 1.5 times the IQR. All other values are considered to be outliers and plotted as individual points

Upon low and high dose macimorelin stimulation, glucose levels were stable around 5 mmol/l and showed no notable dynamics over the 120 min (Fig. 3a, Table 2). Under both dosages, no major change in copeptin was observed, and the median maximum change in copeptin was with values of 0.4 [− 0.5, 0.7] pmol/l and − 0.1 [− 0.5, 0.2] pmol/l close to zero (Fig. 3b, Table 2).

### Correlation between decrease in glucose and increase in copeptin levels

**Fig. 4:**
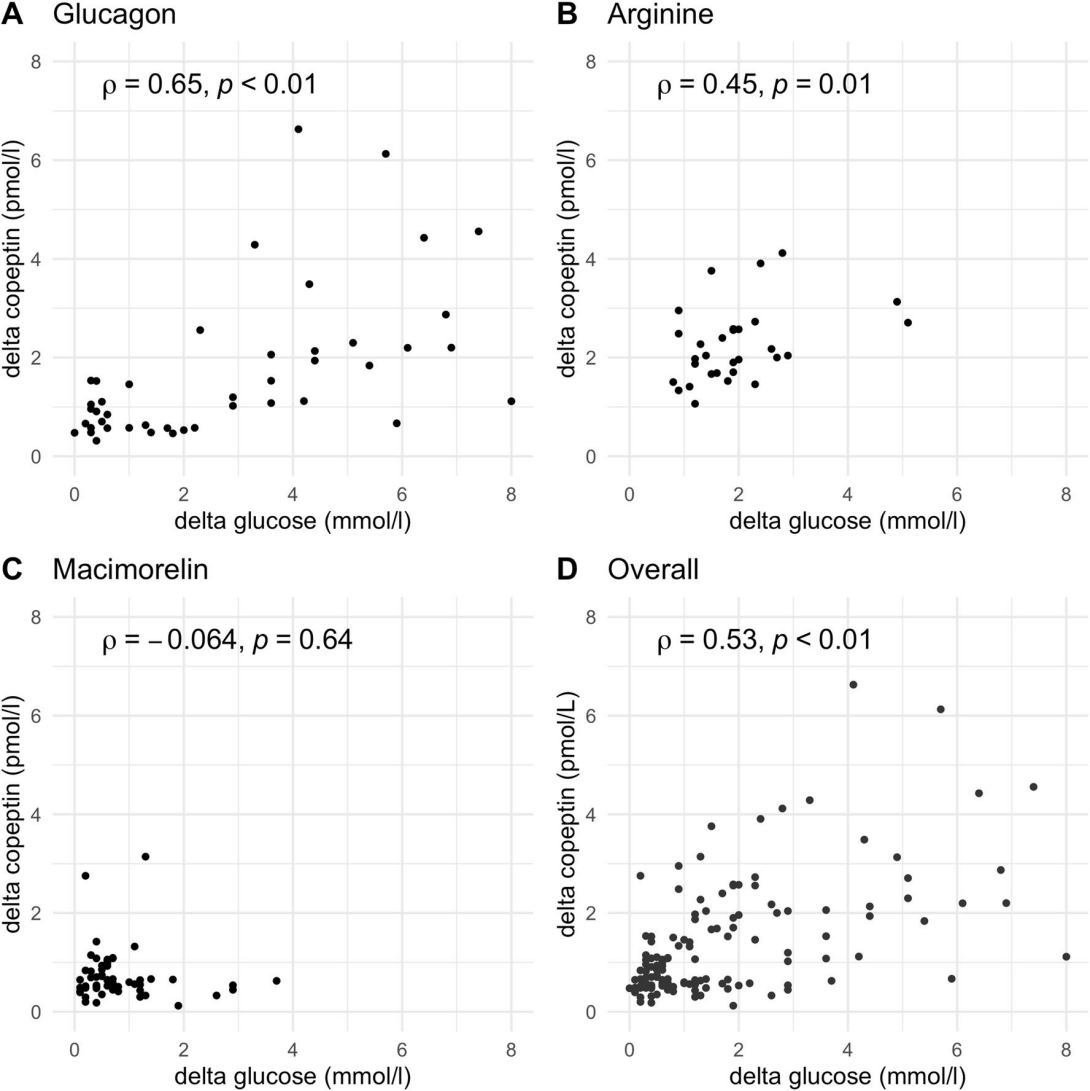
Correlation between glucose and copeptin levels. The delta in the decrease of glucose levels, i.e., maximum glucose—minimum glucose, versus the delta in the increase of copeptin, i.e., maximum copeptin—baseline copeptin, upon glucagon (**A**), arginine (**B**), macimorelin (**C**), and for the pooled data set (**D**). The y-axis is log-transformed for better readability. Spearman’s rank correlation coefficient ρ is given for each test and the pooled data set

In the pooled dataset, a decrease in glucose levels was significantly correlated with copeptin increase (ρ = 0.53, p < 0.01). For each test separately, a strong correlation for the glucagon stimulation (ρ = 0.65, p < 0.01) and a moderate correlation for the arginine stimulation (ρ = 0.45, p = 0.01) was observed. In contrast, no correlation was observed under macimorelin (ρ = − 0.064, p = 0.64) (Fig. 4).

### Growth hormone dynamics upon glucagon and macimorelin stimulation

**Fig. 5:**
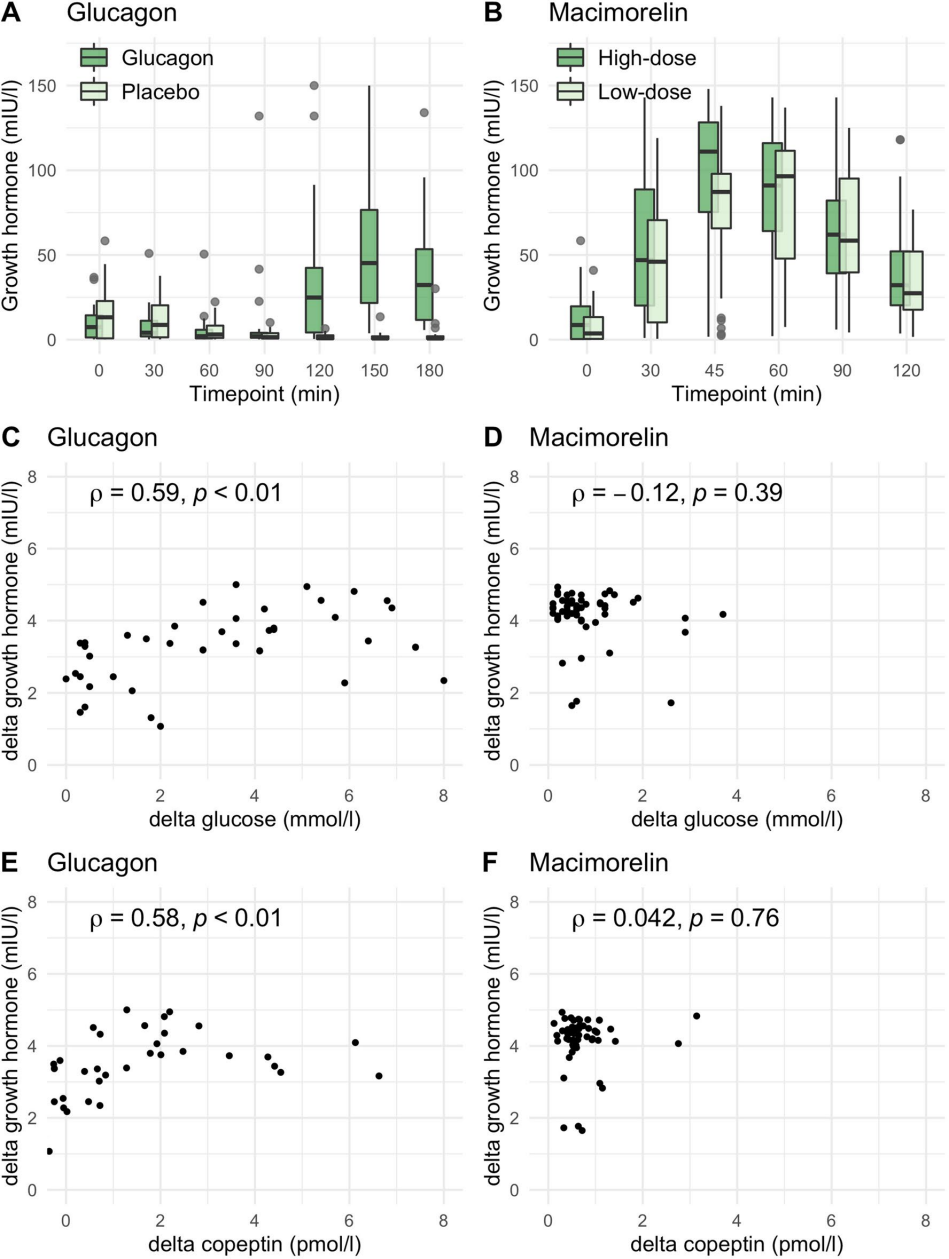
Growth hormone levels upon glucagon and macimorelin. Plasma growth hormone levels in mlU/l after glucagon (**A**) and macimorelin (**B**) stimulation (low dose, i.e., 0.5 g/kg bodyweight; high dose, i.e., 0.75 g/kg body weight). Boxes span the interquartile range [IQR]; the thick horizontal line is the median. Whiskers are the most extreme values lying within the box edge and 1.5 times the IQR. Correlations between delta in the increase of growth hormone upon glucagon and macimorelin versus delta in the decrease of glucose (**C**, **D**) and increase in copeptin levels (**E**, **F**). The y-axis for (**C**–**F**) and the x-axis (**E**, **F**) is log-transformed for better readability

Upon glucagon stimulation, the median [IQR] growth hormone level at baseline was 7 [1, 14] mlU/l and peaked at 45 [22, 77] mlU/l after 150 min (Fig. 5a, Table 2). The increase in growth hormone upon glucagon stimulation correlated with the decrease in glucose levels (ρ = 0.59, p < 0.01) and the increase in copeptin levels (ρ = 0.58, p < 0.01) (Fig. 5, Table 2).

Upon macimorelin stimulation (high dose), the median [IQR] growth hormone level at baseline was 9 [0, 20] mlU/l and peaked at 111 [75, 128] mlU/l after 45 min (Fig. 5b, Table 2). The increase in growth hormone upon macimorelin stimulation showed no correlation with changes in glucose levels (ρ = − 0.12, p = 39) and no correlation with changes in copeptin levels (ρ = 0.042, p = 0.76).

## Discussion

Our study has two main findings. First, we show a similar course in plasma glucose after glucagon and arginine stimulation, while macimorelin had no effect on glucose levels, and second, we show a strong correlation between the decrease in glucose levels and increase in copeptin after glucagon and arginine stimulation.

Arginine vasopressin (AVP) is produced by hypothalamic magnocellular and parvocellular neurosecretory cells projecting to the posterior pituitary [12]. While magnocellular neurons are supposed to be involved in the osmoregulation, parvocellular neurons contain co-packaged AVP and corticotropin releasing hormone (CRH) in their secretory granules and stimulate adrenocorticotropin (ACTH) secretion from the anterior pituitary after non-osmotic stimuli [12, 13]. Hypoglycemia is one of the most potent non-osmotic stimuli for the pituitary gland and induces an acute increase in prolactin, ACTH, growth hormone (GH) [6], and AVP [5]. Specifically, for AVP, Kacheva et al. showed an increase in copeptin after hypoglycemia induced by an insulin tolerance test with maximum copeptin levels after 47 min, while the glucose nadir was at 30 min [14]. Therefore, the insulin-hypoglycemia test can be used to assess anterior and posterior pituitary functions.

Whether a drop in glucose levels itself directly stimulates AVP release or whether this rapid drop triggers multisynaptic stress pathways [15] leading to AVP release remains elusive and cannot be answered with our correlation study. A study in primates suggests that the mammillary nuclei and the lateral hypothalamic nuclei might be glucose-sensing areas, independently from osmolality and epinephrine secretion [16]. The pathway connecting the glucose-sensing nuclei to GH release might include the ventromedial nucleus and the median eminence of the hypothalamus [16]. Our data demonstrate a clear correlation between a decrease in glucose and an increase in copeptin levels upon glucagon and arginine administration. We observed a rapid drop of glucose levels, but no hypoglycemia, which could support the hypothesis of a glucose-drop activating central sensing area rather than hypoglycemia-like stress response.

The glucagon stimulation test is an alternative to the insulin-hypoglycemia test for the evaluation of anterior pituitary function in adults and children [17]. One major advantage is the combined evaluation of ACTH and GH secretion [17]. The exact mechanism of glucagon-induced GH secretion is unclear; however, already in 1975 Mitchell and colleagues suggested a relationship between the rapid drop of elevated glucose levels observed after administration [18]. Glucose course after glucagon injection might mimic an insulin-induced hypoglycemia state, characterized by rapid decrease of glucose levels, but without leading to absolute hypoglycemia. In support of this, the same group demonstrated a suppressed GH peak upon glucagon stimulation if the participants received an additional continuous glucose infusion preventing the rapid drop of glucose levels. We have recently shown a notable increase in copeptin simultaneously with an increase in GH after subcutaneous glucagon injection [11]. One could hypothesize that the increase in GH directly stimulates copeptin release. However, the isolated increase in GH upon macimorelin supports a common stimulus (glucose drop induced stress response) for GH and copeptin upon glucagon rather than a direct stimulation.

Arginine might act in two possible synergistic ways, in the first part of the test via direct central hypothalamic/pituitary stimulation, and second half of the test via the change in glucose levels. In support of this, a continuous increase of copeptin was observed within the first 30 min after arginine infusion, whereas the increase of copeptin after glucagon injection was only observed after glucose nadir. More precisely, L-arginine conversion into L-citrulline is catalyzed by nitric oxide synthases (NOS) located in many cells including hypothalamic AVP producing neurons with nitric oxide (NO) as the product [19]. In vivo studies have proposed a direct stimulatory effect of NO on AVP release [20]. Interestingly, administration of NOS inhibitors to rats induced a mild polyuria-polydipsia syndrome probably due to deficient AVP secretion [20]. In contrast, the exogenous administration of a NO donor, i.e., arginine, could explain the direct acute release of AVP.

Glucose course after arginine infusion was very similar to the one observed after glucagon injection, i.e., an initial increase followed by a rapid decrease to low-normal levels, although with a smaller drop (delta) in glucose. Arginine leads to depolarization of pancreatic beta cells and consequently voltage-dependent calcium channels, which stimulates insulin secretion [21], possibly explaining the later drop in glucose. Besides its direct central effect, arginine might stimulate AVP in addition via drop in glucose levels explaining the later constant plateau of copeptin in the second half of the test.

Macimorelin, a synthetic ghrelin receptor agonist, was recently proposed as a simplified test in the diagnosis of GH deficiency [8]. It is characterized by rapid absorption after oral intake with a plasma peak concentration of around 60 min [22], at which a GH peak is observed in healthy adults. We recently showed no effect on copeptin levels and now show that there is no effect on glucose levels. Macimorelin might act directly on hypothalamic and pituitary ghrelin receptors, leading to a direct and selective GH release or modulation of growth hormone releasing hormone (GHRH) [23]. Both hypothalamus and pituitary are potential sites of action; in animal models, hypothalamic-pituitary disconnection led to decreased GH release after administration of ghrelin receptor agonists supporting the hypothesis of hypothalamic receptor action[24]. Conversely, pituitary cell cultures show a marked increase of GH after incubation with ghrelin receptor agonists [25]. We show a GH peak between 45 and 60 min after intake of macimorelin. This increase in GH did not correlate with either glucose or copeptin changes, which supports our hypothesis of a glucose-independent stimulation of GH upon macimorelin. In summary, we assume that macimorelin selectively stimulates GH release via specific receptors rather than a direct glucose-, a secondary glucagon- or stress-induced GH and AVP/copeptin secretion.

Our study has limitations. The main limitation of our analysis is that we could not measure plasma insulin and glucagon levels, because the preanalytical requirements were not met. Second, this is a post-hoc analysis of three prospective studies.

In conclusion, this study shows a correlation between the drop in glucose levels and the increase in copeptin levels upon arginine and glucagon stimulation. In contrast, upon macimorelin stimulation, glucose and copeptin levels remain unchanged. These findings suggest that a drop in glucose levels might be a possible explanation for the increase in copeptin levels in the first two stimulation tests. Whether the drop in glucose levels itself leads to copeptin secretion, or whether it mirrors other stimuli such as hypoglycemia-induced stress cannot be answered with our study and could be better defined in further research measuring glucagon and insulin levels throughout the stimulation tests.
